# Using measures of wellbeing for impact evaluation: Proof of concept developed with an Indigenous community undertaking land management programs in northern Australia

**DOI:** 10.1007/s13280-018-1058-3

**Published:** 2018-05-05

**Authors:** Silva Larson, Natalie Stoeckl, Diane Jarvis, Jane Addison, Sharon Prior, Michelle Esparon

**Affiliations:** 1 Division of Tropical Environments and Societies, James Cook University, Building 1, Townsville, QLD 4811 Australia; 2 College of Business, Law and Governance, James Cook University, Room 129, Building 145, Townsville, QLD 4811 Australia; 3 CSIRO Land and Water, Townsville, Australia; 4 College of Business, Law and Governance, James Cook University, Room 122, Building 145, Townsville, QLD 4811 Australia; 5 Ewamian Aboriginal Corporation, 9 Hort Street, Mareeba, QLD 4880 Australia

**Keywords:** Impact evaluation, Indigenous land management, Life satisfaction, Monitoring and evaluation, Subjective wellbeing, Theory of Change

## Abstract

Combining insights from literature on the Theory of Change, Impact Evaluation, and Wellbeing, we develop a novel approach to assessing impacts. Intended beneficiaries identify and rate factors that are important to their wellbeing, their satisfaction with those factors now, and before an intervention. Qualitative responses to questions about perceived changes and causes of change are linked to quantitative data to draw inferences about the existence and/or importance of impact(s). We use data from 67 Ewamian people, in a case study relating to Indigenous land management, to provide proof of concept. ‘Knowing that country is being looked after’ and ‘Having legal right/access to the country’ were identified as important to wellbeing, with perceptions that Native Title determination, declared Indigenous Protected Area and associated land management programs have had a significant and positive impact on them. Further method testing might determine the utility of this approach in a wide range of settings.

## Introduction

Impact evaluation (IE) can be roughly defined as the field of evaluative practices aimed at assessing the effects of various interventions/activities (Vaessen 2010; Stern et al. 2012). The OECD-DAC (2002, p. 24) defines impacts as the positive and negative; primary and secondary; direct or indirect; intended or unintended, results of an action. In this paper, we propose, and then demonstrate, a novel way of evaluating diverse impacts from activities and programs related to the natural environment, on human society and individuals. We bring together ideas from the “Theory of Change” (ToC) and insights from literature relating to human wellbeing, to develop our approach.

A perceived causal relationship—what has caused the perceived impact—is at the heart of the proposed method, and is captured in both a qualitative and quantitative manner. The method uses a point-based system that can equally measure all types of perceived benefits and costs (monetary and non-monetary). Since the perceived impacts are elicited directly from intended beneficiaries, the method is not limited to assessing only the positive, primary, intended and prescribed impacts identified by the evaluator; but rather has the potential to capture a whole range of perceived impacts including negative, secondary, indirect and unintended ones.

We use data from a case study relating to Australian Indigenous Land and Sea Management Programs (ILSMPs), to provide proof of concept. Australian Indigenous people have managed their country for tens of thousands of years, undertaking a variety of different traditional land management practices. These practices involve much more than just managing the physical environment; Indigenous people also seek to manage the values, resources, stories and cultural obligations associated with a geographical area (Hill et al. 2013). Evaluation of the impact of ILSMPs is exceedingly difficult because of the numerous interacting relationships between environmental condition, individual and community wellbeing and the role of so-called “co-benefits”—a diverse range of benefits that reach far and above those associated with the environment and that accrue to a wide and diverse range of stakeholders. Reducing uncertainty and complexity in the identification, evaluation, and monitoring of such co-benefits is emerging as a research priority (Barber and Jackson 2017). Failure to properly account for some of the more *complex* benefits associated with ILSMPs may lead to their degradation or loss (Stoeckl et al. 2018).

The conceptual framework used in the development of the method is presented first, followed by a description of an empirical application of the method, a case study of changes discussed by 67 Ewamian people in Queensland Australia relating to their recent Native Title (NT) determination, declared Indigenous Protected Area (IPA) and associated ILSMPs. A discussion of our learnings and points for further research is presented in closing.

## Theoretical overview

Our conceptual framework blends insights from the literature on human wellbeing, the ToC and participatory (evaluative) methods. ‘Wellbeing’ is a holistic concept with both subjective and objective dimensions relating to people’s overall quality of life and the factors affecting it (Diener et al. 1999; Stutzer and Frey 2010). The pathways that lead to wellbeing are thus complex, and, as highlighted in the ToC approach, there is a need to (a) clarify and describe the causal assumptions behind pathways towards wellbeing (Mayne and Johnson 2015) and (b) ‘demonstrate contribution’ rather than ‘prove causality’ (Wimbush et al. 2012). This is particularly the case when the impacts of an activity/program on wellbeing, such as those relating to environmental management, are assessed. The subjective nature of some aspects of wellbeing also means that those for whom wellbeing is being assessed must be participants in the assessment and mapping of causal assumptions and its proximate pathways, as well as in the demonstration of contribution. Each of the three bodies of literature that inspired our conceptual approach, human wellbeing; the ToC; and participatory (evaluative) methods, are further introduced in this section.

The phrase ‘subjective wellbeing’ is often used when referring to people’s own (subjective) evaluations of their lives and is frequently measured by asking people to respond to questions about their satisfaction with life overall and with various factors likely to influence it. A substantial number of researchers have collected data on life satisfaction (LS) and on factors thought to influence it, using statistical techniques to explore the strength of relationships (Diener et al. 1999; Stutzer and Frey 2010; Larson et al. 2015; Chacon et al. 2016; Jarvis et al. 2017). Numerous factors have been found to influence LS (see Jarvis et al. 2016 for tabulated summary) the critical message being that wellbeing is multifaceted and that LS studies should consider factors from several different ‘life domains’.

The literature on methods for assessing wellbeing also highlights that to gain deeper understanding, one should not only consider people’s satisfaction with various factors but should elicit information about how important they feel those factors are to their overall LS (Max-Neef 1991; Sen 1999; Larson 2011). In line with the philosophy underscoring participatory methods of IE, subjective approaches to the analysis of wellbeing thus take into account individual experiences and have the capacity to help practitioners understand and communicate interpretations, priorities and needs of the people (Diener and Suh 1997).

The ToC (Mayne and Stern 2013; Mayne and Johnson 2015) is one approach to IE that explores the ideas and beliefs people have—consciously or not—about why and how the world and people change, including the proximate causes of their own wellbeing. VanEs et al. (2015) argue that empirical applications of the ToC are lacking in methods that (a) can allow for the differing assumptions of different stakeholders about what worked, why and by how much, (b) can capture and measure both monetary and non-monetary benefits (and costs) on an equal footing, and (c) have the capacity to capture both the intended and the unintended effects of an intervention/change (Mayne and Stern 2013). Our method aims to fill some of these gaps by providing evidence of contribution (sometimes referred to as *attribution*). We do this by explicitly asking beneficiaries what they perceive to have caused the change in their wellbeing, thus minimising risk of misunderstanding their motivations and objectives (Barber and Jackson 2017). Combining qualitative and quantitative responses further allows for understanding the extent and significance of change.

Our method reflects the underpinning philosophy that participation in the assessment and causal mapping of wellbeing is crucially important because people are not passive recipients of opportunities to improve their health, wealth and social standing offered through various initiatives: context is key to understanding the interplay between activities and their effects (Blamey and Mackenzie 2007). Importantly, participation can lead to creation of new knowledge, shared understanding, trust and collective action (Pahl-Wostl 2006; Lebel et al. 2010; Hegger et al. 2012). Context itself is multifaceted and operates at a variety of levels. How people perceive and understand change and the world around them are based on personal beliefs and assumptions. Beliefs are formed in complex ways, argue vanEs et al. (2015), with numerous contributing and mediating factors, including: socioeconomic status, age and gender; education; personal experiences; and the culture in which one lives. Within this understanding, participants learn, have ‘agency’ and can help ‘cause’ successful outcomes through their own actions and decisions (Stern et al. 2012); they can likewise contribute to unsuccessful outcomes.

Participatory approaches explicitly set out to include a diversity of participant views in study design. Each participant group (impact according to whom?) is given an opportunity to describe their perceptions of both what creates an impact (the impact of what?) and what was affected (impact on what?) (Vaessen 2010). Creswell and Miller (2000), based on Schwandt (1997), define validity as *how accurately the account represents participants*’ *realities of the social phenomena and is credible to them.* In this context, it is the validity of inferences drawn from data, not the data itself, that matters most. It is also important to think carefully about *whose* perspectives matter in assessment of ‘validity’, since different participants (e.g. government, community, researchers) will likely have different interpretations and perspectives of social phenomena. Rather than excluding stakeholders from study design and omitting impacts that cannot be easily quantified in monetary terms, hybrid participatory valuation approaches, which allow for stakeholder input (Delisle 2013; Stoeckl et al. 2014; Larson et al. 2015; Jarvis et al. 2017) are increasingly being used.

## Approach

### Methods

**Fig. 1:**
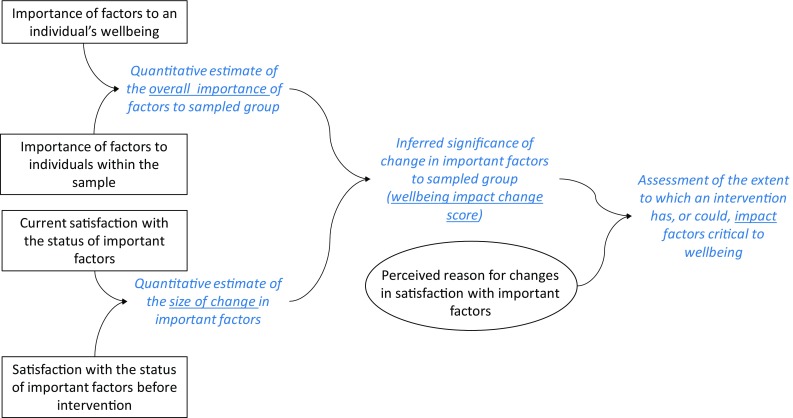
Conceptual framework for our proposed Wellbeing-based method for Impact Evaluation approach (W-IE). Information elicited directly from intended program beneficiaries shown in boxes (quantitative data) and ellipse (qualitative data), information inferred from responses to direct questions shown in italics (without frame)

We use insights from the above literatures to propose a method (conceptualised in Fig. 1) for evaluating the wellbeing impacts of activities and programs, such as those related to improving environmental condition.

First, we suggest that one should ask individual participants to identify factors that are important to their wellbeing and to provide a subjective importance score for each identified factor. One can then use individual responses to generate a ‘whole of sample’ score for each factor, by multiplying each factor’s importance score by the percentage of individuals within a sample who identified that factor as important. Critically, the way this indicator is constructed imposes an implicit assumption on the data, namely that the wellbeing of the sample (or community) is a simple function of the wellbeing of individuals within it—an assumption that is common to other assessment methods (such as cost–benefit analysis), but which is not uncontroversial.

Second, we suggest that one should assess people’s satisfaction with core factors—both now and previously (before activity/program occurred). Subtracting one satisfaction score from the other generates a quantitative measure of perceived change. Information about the magnitude of perceived change can be combined with sample importance scores, to draw inferences about the significance of perceived change to the wellbeing of the participant group being assessed.

Third, we suggest that one should ask people about their subjective perceptions of the reasons for observed change. These qualitative responses can be combined with information about the significance of perceived changes, to draw inferences about the extent and importance of an activity/program’s impact (Fig. 1).

### Case study description

**Fig. 2:**
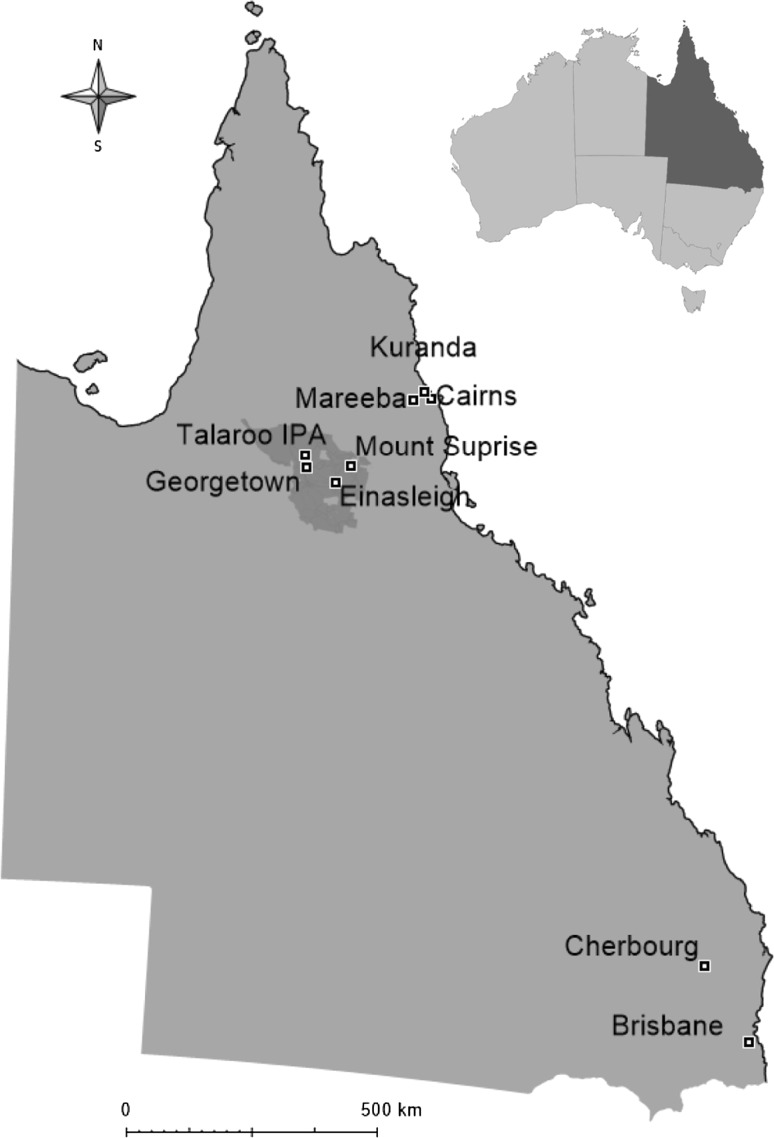
Map showing approximate location of the 2013 Declared Native Title boundary of the Ewamian people traditional lands; and towns/centres in which there are relatively large populations of Ewamian people living now

The Ewamian people originate from the Agwamin Society with traditional lands in the Einasleigh Uplands region, inland from Cairns, North Queensland (Fig. 2). During the late nineteenth century, as a result of European colonisation and government policies, the Ewamian people were disposed of their lands. Although many remained in the general area, some living at the Georgetown Reserve and many employed as stockman and domestic help through to the 1980s, there are also Ewamian people living throughout Queensland with significant populations in North Queensland and Brisbane and Cherbourg (Fig. 2).

The Ewamian Aboriginal Corporation (EAC) was registered in 1994, to support an application for NT (the process began in 1997).^1^ In 2012, The Indigenous Land Council acquired Talaroo Station (31 500 ha with much pastoral land, and significant cultural and strategic values), with EAC as lessee. In 2013, NT applications were successfully determined for more than 29 000 square kilometres of Ewamian land, and in 2017, the Deeds of Talaroo Station were transferred to EAC on a 30 Year Rolling Term Lease. In 2018, Talaroo will also officially be declared as an IPA by the Department of Prime Minister and Cabinet (DPMC). EAC employs a ranger coordinator and 4 full time rangers [funded by the Queensland Indigenous Land and Sea Ranger Program (DES)] to manage Talaroo with funding assistance also received through the IPAs Program (DPMC).

Our primary interest here, is the extent to which the various ILSMPs in which the EAC are involved are impacting the wellbeing, or factors important to wellbeing, of the Ewamian people.

When developing our questionnaire, we first needed to select a finite set of factors that potentially contribute to one’s wellbeing. We started with a review of literature relating to Indigenous wellbeing and developed an initial list of 45 wellbeing factors. After a series of consultations with other researchers and with members of the Ewamian Board, this list was reduced to 25 wellbeing factors, tested in our study (Table 1). Each factor was presented to respondents as cards with words and images.

**Table 1:** Final 25 wellbeing factors tested in the study

Wellbeing factors	Referred to in the paper as
Having enough power to influence decisions that affect my life (e.g. decisions about housing, about how to spend money, etc.)	Decision-making
Being a role model or having role models in the community	Role model
Having the legal right to use/access country	Access to country
Knowing that country is being looked after the right way	Country looked after
Being out on country (for any reason)	Being on country
Obtaining legal protection for places, knowledge or practices with important cultural value	Legal protection
Feeling strong in our culture	Strong in culture
Making sure language is not ‘lost’ (spoken regularly and/or written down)	Language
Sharing knowledge (traditional and new) within and outside community	Sharing knowledge
Having houses that are in good condition and not overcrowded	Housing
Having good quality schools and training centres close by	Schools
Having good quality clinics and hospitals close by	Health centres
Reducing how much I use grog, smokes or gunja	Social ills
Feeling good and strong in my body and mind	Strong person
Knowing my family are feeling good and strong in their bodies and mind	Strong family
Knowing that people in our community feel good about each other and work together to help when needed	Community spirit
Knowing that my community is a safe place for me and my loved ones	Safe community
Knowing that people who behave outside the law (or Aboriginal law) are punished	Law enforced
Having a paid job	Paid job
Enjoying the work I do (paid or unpaid)	Work satisfaction
Having more money	More money
Having my own business	Own business
Being able to save money for big purchases (e.g. car or house)	More saving
Having jobs available in my local community	Local jobs
Being able to use a mobile phone and internet in our community and on country	Communication

Based on our conceptual framework (Fig. 1), we constructed a questionnaire to elicit the following key issues:

- Importance of different factors known to contribute to wellbeing: we asked respondents to tell us, on a scale of 1–10, how important the factor is to their wellbeing;
- Perceptions of change in each of those factors were determined by asking respondents to tell us their satisfaction with each important factor (a) now, and also (b) 5 years previously (a period approximately preceding granting of NT and consequent IPA declaration);
- Whenever change was noted, we asked what had happened to cause the change, qualitatively exploring (without prompting) if any changes were attributed to, or associated with, NT, the granting of the IPA and associated ILSMPs.

A copy of the questionnaire is available from authors on request.

Interviews were conducted face-to-face by a research team that included at least one Ewamian research assistant between April and June 2017. A total of 67 Ewamian people were surveyed, of which 37% were males. The age of respondents ranged from 17 to 79, with a median of 47. All respondents spoke English at home. Only two respondents lived ‘on country’ (traditional lands), 52% lived in North Queensland in the relative vicinity of traditional country and 48% lived more than 1000 km south (Brisbane/Cherbourg area, Fig. 2). Employment was the main source of income for 27% of respondents only.

## Results

### Overall importance of wellbeing factors

Each factor presented in the survey was selected as being critically important by at least one respondent, indicating that our selection process had allowed for the identification of factors relevant to respondents.

Table 2 lists the factors selected most frequently as being the most important. The first column shows the quantitative estimate of the overall importance of those factors. This estimate was calculated by multiplying the average importance score assigned by respondents with the percentage of respondents selecting each factor (numbers in brackets). This was done because some factors may be considered important by most people, others may be critically important to a smaller sub-group (with the first example having a higher % selecting, and the second having a higher mean importance score). Health centres, paid jobs, access to country, safe community and role models emerged as the most important factors to the largest percentage of respondents (Table 2).

**Table 2:** Wellbeing factors with the highest overall importance (largest numbers of respondents reporting high importance for the factor), with the reported size of change in satisfaction (the difference in satisfaction scores between now and 5 years ago)

Wellbeing factor (top 10 based on overall importance)	Overall importance to sample (importance score × % selecting)	Size of change (current satisfaction score − past satisfaction)	Wellbeing impact change score (overall importance × size of change)
Health centres	4.00 (9.57 × 42)	0.44 (9.07–8.63)	1.76
Paid job	3.98 (9.52 × 42)	0.09 (7.37–7.28)	0.35
Access to country	3.68 (9.48 × 39)	1.97 (7.88–5.91)	7.25
Safe community	3.59 (9.63 × 37)	0.00 (7.67–7.67)	0
Role model	3.46 (9.65 × 36)	1.22 (8.31–7.09)	4.22
Strong family	3.15 (9.59 × 33)	0.64 (7.82–7.18)	2.02
Strong in culture	3.10 (9.45 × 33)	0.91 (8.95–8.04)	2.82
*Local jobs*	*3.04* (*9.27* × *33*)	− *1.45* (*4.95*–*6.40*)	− *4.41*
Country looked after	2.94 (9.38 × 31)	2.95 (9.19–6.19)	8.67
Strong person	2.55 (9.50 × 27)	1.17 (8.44–7.28)	2.98

*Size of change*  < 1 = average, 1–2 = high,  > 2 = very high, *Wellbeing Impact change score* < 1 = –, 1–4 = high, > 4 = very high

Italic value indicates the wellbeing factor received negative change score

### Changes in wellbeing

Estimates of the size of change in factors important to wellbeing are presented in the second column of Table 2, calculated as the difference between reported current and past satisfaction with the factor. Over the last 5 years, increases in satisfaction have been recorded for all but two factors, local jobs (decrease in satisfaction of 1.45 points) and the prevalence of social ills (decrease of 0.78 points, not selected as of very high importance and hence not included in Table 2). Not only did ‘local jobs’ record the highest decrease in satisfaction, it already had a very low satisfaction scores of 6.40, bringing it down to the lowest satisfaction of all factors, at only 4.95 points.

Out of the most important wellbeing factors, large increases in satisfaction were recorded for ‘country being looked after’ (an average 2.95 point increase) and ‘access to country’ (1.97). ‘Owning a business’ and ‘language’ also recorded very high increases in satisfaction (4.33 and 3.18 point increase, respectively), but these were selected as being of importance by a limited number of respondents only and hence are not included in Table 2.

### Reasons for change in wellbeing

The largest increases in reported satisfaction with the most important factors were those relating to ‘Country looked after’ (mean improvement of 2.95) and ‘Access to country’ (1.97, Table 2). A qualitative exploration of responses to open-ended questions about perceived reasons for change in these two factors revealed clear perceived linkages between those positive changes and (a) ILSMPs, specifically ranger programs, and (b) the NT/IPA processes (respondents did not always clearly differentiate between NT and IPA processes). The following reasons were, for example, given to explain increased satisfaction with ‘knowing that the country is being looked after’:

I didn’t know the country back then. Knowing there’s rangers there is good, and there’s old people there. We know the spring is being looked after, and there are no cattle there now so the grass is good. Paraphrased from R12

People are now looking after land, we have rangers etc. Paraphrased from R23

We are fortunate to have a ranger program which has people on land looking after it the right way. The elders fought hard for what we have now, now we need to look after it. R30

So ILSMPs in general, and the ranger program in particular, appear to be well recognised and regarded as having a significant, positive impact on factors deemed crucially important to wellbeing. Similar attributions were recorded in relation to ‘Access to country” (satisfaction increase of 1.97 points), for example,

We didn’t have any access to land 5 years ago Paraphrased from R4

Native Title determination has improved access R8

It’s improved because people know more about where their areas actually are …The process of getting Native Title taught us about our traditional areas. R17

However, it is important to note that most of those interviewed had not visited Talaroo Station (the declared IPA). Comments about access thus reflect ability (legal right) to access, and do not require a physical visit.

Further, positive change in important wellbeing factors not directly related to ‘country’ was also attributed to the NT/IPA processes. For example, in relation to ‘Strong person’ (positive change of 1.17 points), respondents reported,

Now we have our land I can tell my family and feel better and share that. R2

On the other hand, there were no perceived linkages reported by any of the respondents between NT/IPA or ranger programs, and availability of local jobs. The ranger program was mentioned several times as a positive development, in relation to the country being looked after well. But comments related to local jobs were associated with recent losses of mining jobs (due to economic/resource downturn) and loss of agricultural jobs (attributed to change in the federal ‘working visa’ program that increases the ease by which foreigners—international backpackers—to work as agricultural labourers), with little mention of the ranger program as an employment opportunity—although a few mentioned aspirations to have a job as a ranger in the future. This likely reflects the fact that the number of jobs provided by the ranger program (five positions) is small relative to the size of the towns and economies in which these programs operate.

### The impact of the program on wellbeing

**Fig. 3:**
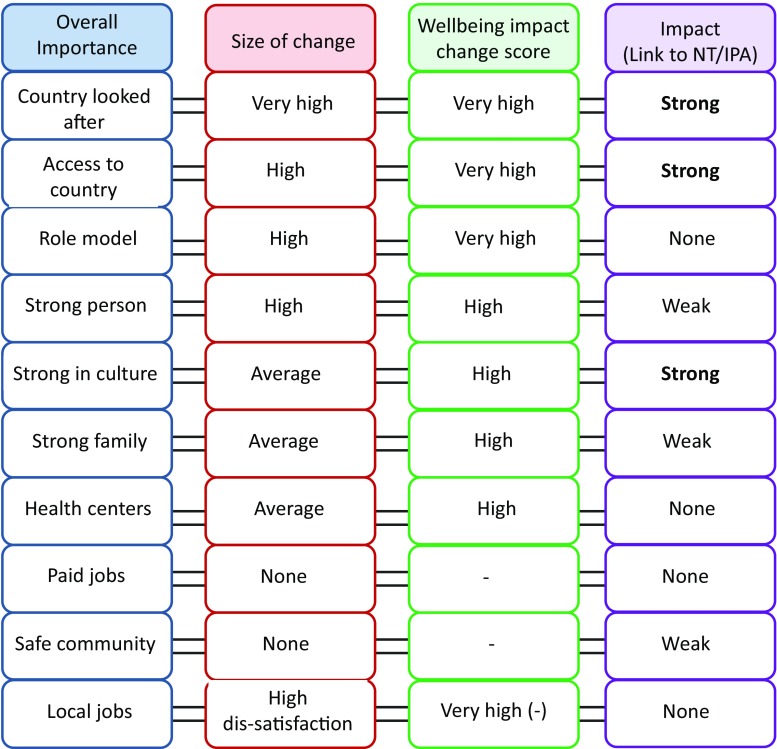
Populating conceptual W-IE framework: information elicited directly from intended program beneficiaries was used to estimate the overall importance of each wellbeing factor and the size of change in satisfaction. Those two parameters were then used to infer the ‘wellbeing impact change score’; while qualitative data elicited from respondents allowed us to report linkages (or lack thereof) between the NT/IPA processes and the change in satisfaction, as perceived by the respondents

We used quantitative data collected from the respondents to estimate the overall importance and size of change for each wellbeing factor (Table 2; Fig. 3). Combining those two parameters allowed us to infer the significance of change, in terms of its potential ability to impact overall wellbeing. Qualitative data on perceived reasons for change in satisfaction with the wellbeing factors, some of which are presented in the previous section, provided specific ‘theories’ of causes of positive and negative changes in LS (Fig. 3). This also allowed us to gauge if the perceived linkages to NT/IPA processes were recognised, and if so, what the strength of the link was. The strength of linkage was determined based on percentage of respondents selecting the factor, who reported the NT/IPA processes as a cause of the recorded impact. We defined these linkages as ‘weak’ if less than 30% of respondents mentioned the link, ‘strong’ if 30–70% of respondents mentioned the link and ‘very strong’ if link was mentioned by more than 70% of respondents (Fig. 3). This determination of strength was somewhat arbitrary and would need further refinement in future studies.

## Discussion

In this paper, we focus our attention on the evaluation of impacts on people, leading from activities/programs related to the natural environment. After highlighting significant gaps in the literature, we conceptualised a wellbeing-based method for conducting IEs (W-IE). In addition to presenting the conceptual framework for this mixed-method quantitative and qualitative participatory technique, we provided an empirical proof of concept, based on a case study with Ewamian people involved in various indigenous land management programs in North Queensland, Australia. We propose that this approach could be used for IE of programs with indigenous cultures in other parts of the world. Indeed, the general approach could be used for IE of programs in any culture, throughout the world. The actual lists of wellbeing factors to be included in the study (Table 1) should be modified for each specific setting; however, the general methodological approach could be replicable. Further, with minor contextual modifications to the process itself, we argue the approach could potentially be used on a whole range of social–ecological programs around the globe, in particular development aid programs, environmental initiatives and educational/awareness programs.

To understand how and why an activity is (or is not) working, there is a need to understand how the activities of the program are expected to lead to the desired results, and why the various links along the way are expected to work (Mayne and Johnson 2015). This method specifically aimed to help with respect to (a) delimitation, (b) attribution and causality, (c) measurement of both monetary and non-monetary impacts on equal footing, (d) capturing of both positive and negative changes, and (e) capturing of unintended impacts.

The criteria for delimitation in IE concern the questions of ‘impact according to whom’, that is, which types of processes of change and effects are valued as important and by whom. Using the approach described in this paper, we collected perceptions of impact directly from the intended beneficiaries. The scoring of the importance of a range of factors by beneficiaries, allowed us to understand the relative importance of each factor, and hence the importance of related impacts. Factors related to ‘country’, which received very high importance scores, were perceived as strongly linked to ILSMPs (ranger programs and the IPA) and to NT, suggesting that changes due to these, related programs, are valued as highly important by the beneficiaries themselves. As work by Denham (2017) found elsewhere, attention to indigenous sovereignty and self-determination in program implementation contributes to widely appreciated socio-environmental benefits. In this case, the method was used to capture perceptions of beneficiaries themselves, but could also be used to capture perceived benefits, costs and linkages of policy makers, land managers, pastoralists and other stakeholders.

Human wellbeing and LS approaches provide a valuable alternative to more traditional dollar-denominated methods, as they allow for the measurement of both monetary and non-monetary impacts on equal footing (Larson 2011). Non-monetary impacts may be just as significant as monetary impacts—perhaps even more (Soderbaum 2013). Scientists and evaluators must therefore be careful not to focus only on easily measurable biophysical or economic metrics and exclude considerations that really matter to people (Satz et al. 2013). Exclusion by omission may negatively impact the very things one is trying to protect or improve (Stoeckl et al. 2018).

The capacity of our method to capture both positive and negative changes was also demonstrated in this proof of concept study. Two factors, ‘local jobs’ and ‘social ills’, received negative satisfaction scores (i.e. current satisfaction was reported as lower than it was 5 years ago before programs started). Although neither of the negative changes reported were linked to the ILSMPs/NT/IPA programs, the programs also did not alleviate the negative impacts created by other economic and social processes.

What could be termed an ‘unexpected impact’ was also captured during the study, as the (perceived) positive impact of NT/IPA on housing. One respondent suggested that the granting of the NT and IPA processes resulted not only in legal recognition of Ewamian people, but also in increased respect of the wider community towards them (an impact also identified by SVA 2016). This respondent felt that the added respect, among other things, evidenced itself in perceived decreased discrimination in a range of life activities, including less discrimination when accessing housing. This linkage is very weak as it was based on perceptions of one respondent only, and should not be interpreted as ‘proof’ that NT/IPA will improve housing. But this does clearly emphasise the power of our approach to identify ‘unexpected’ impacts (which, if interesting or important enough, could be investigated further in a follow-up study).

## Conclusion

In this paper, we combine insights from literature on the ToC, IE and Wellbeing, to develop a novel approach to assessing impacts of an activity/program. The approach asks intended beneficiaries to identify and rate factors that are important to their wellbeing, also rating their satisfaction with those factors. Qualitative responses to questions about perceived changes and causes of change in satisfaction with those factors are recorded. Because factors, types of and causes of change are all elicited from program beneficiaries, they are not limited to including only impacts pre-conceived by evaluators, but can capture a whole range of positive and negative, unexpected and unintended impacts.

‘Knowing that country is being looked after the right way’ and ‘Having legal right/access to the country’ were identified as very important to wellbeing by the largest percentage of the respondents from the Ewamian people in North Queensland. Those two factors, plus ‘Feeling strong in our culture’, were also the factors most strongly linked to the NT/IPA processes and ILSMPs. The overall perception was that the recently declared IPA and its associated NT determination had had a significant and positive impact on them.

Further method testing might determine its utility across wide range of settings. We propose that this approach could be used for IE of not only programs related to indigenous cultures, but also for a whole range of social–ecological or livelihoods-related programs around the globe.

Our ‘proof of concept’ trial highlights the potential of our method as an evaluative tool. With further development and refinement, it could prove a valuable addition to our existing toolbox of evaluative methods—generating additional insights into the impact of an activity/program in a wide variety of contexts that include, but are not limited to: development aid programs, environmental initiatives and educational/awareness programs.

## Footnotes

1 The *Native Title Act* (*Act No. 110*) *of 1993* establishes legal framework for the protection and recognition of Aboriginal and Torres Strait peoples’ Native Title (NT) and sets up processes to determine where NT exists, how future activity impacting upon it may be undertaken, and to provide compensation where it is impaired or extinguished.
