# Correction to: Using measures of wellbeing for impact evaluation: Proof of concept developed with an Indigenous community undertaking land management programs in northern Australia

**DOI:** 10.1007/s13280-018-1113-0

**Published:** 2018-11-02

**Authors:** Silva Larson, Natalie Stoeckl, Diane Jarvis, Jane Addison, Sharon Prior, Michelle Esparon

**Affiliations:** 1 Division of Tropical Environments and Societies, James Cook University, Building 1, Townsville, QLD 4811 Australia; 2 College of Business, Law and Governance, James Cook University, Room 129, Building 145, Townsville, QLD 4811 Australia; 3 CSIRO Land and Water, Townsville, Australia; 4 College of Business, Law and Governance, James Cook University, Room 122, Building 145, Townsville, QLD 4811 Australia; 5 Ewamian Aboriginal Corporation, 9 Hort Street, Mareeba, QLD 4880 Australia

## Correction to: Ambio 10.1007/s13280-018-1058-3

The article “Using measures of wellbeing for impact evaluation: Proof of concept developed with an Indigenous community undertaking land management programs in northern Australia” written by “Silva Larson, Natalie Stoeckl, Diane Jarvis, Jane Addison, Sharon Prior and Michelle Esparon” was originally published electronically on the publisher’s internet portal (currently SpringerLink) on 05 May 2018 without open access.

With the author(s)’ decision to opt for Open Choice the copyright of the article changed on 1 November 2018 to © The Author(s) 2018 and the article is forthwith distributed under the terms of the Creative Commons Attribution 4.0 International License (http://creativecommons.org/licenses/by/4.0/), which permits use, duplication, adaptation, distribution and reproduction in any medium or format, as long as you give appropriate credit to the original author(s) and the source, provide a link to the Creative Commons license and indicate if changes were made.

The original article has been corrected.

